# Synergistic Cardiopulmonary Protection of Endothelin Receptor Antagonists Combined With Soluble Guanylate Cyclase Agonists in High-Risk Coronary Syndrome With Pulmonary Hypertension

**DOI:** 10.31083/RCM46401

**Published:** 2026-03-16

**Authors:** Liyue Zhao, Xinli Pang

**Affiliations:** ^1^Department of Cardiology, The Second Affiliated Hospital of Guangzhou Medical University, 511436 Guangzhou, Guangdong, China; ^2^Department of Cardiology, Shenzhen People's Hospital, 518001 Shenzhen, Guangdong, China

**Keywords:** ambrisentan, riociguat, pulmonary hypertension, high-risk coronary syndrome

## Abstract

**Background:**

The prognosis and long-term survival of high-risk coronary syndrome patients with pulmonary hypertension (PH) remain unsatisfactory, and limited research has evaluated the synergistic therapeutic effects of endothelin receptor antagonists (ERAs) combined with soluble guanylate cyclase agonists (sGCAs). This study aimed to assess the synergistic cardiopulmonary protective effects and clinical safety of ERA combined with sGCA therapy in patients with high-risk coronary syndrome complicated by PH.

**Methods:**

This retrospective controlled study included 132 patients with high-risk coronary syndrome and PH who were admitted between January 2019 and December 2023. After exclusion criteria were applied, 119 patients were analyzed and categorized into a control group (ambrisentan monotherapy, n = 58) and an experimental group (ambrisentan plus riociguat, n = 61) according to the associated treatment strategy. Primary endpoints included 6-minute walk distance (6MWD), N-terminal pro-B-type natriuretic peptide (NT-proBNP) levels, and the World Health Organization-related functional class (WHO-FC). Secondary endpoints included cardiac index (CI), left ventricular end-diastolic diameter (LVEDD), tricuspid annular plane systolic excursion (TAPSE), mean pulmonary artery pressure (mPAP), pulmonary vascular resistance (PVR), Borg dyspnea score (BDS), and the incidence of adverse events.

**Results:**

Baseline characteristics between the two groups were comparable (all *p* > 0.05). Following treatment, the 6MWD, CI, and TAPSE values significantly improved in both groups (all *p* < 0.05), with greater improvements observed in the experimental group (95% CI: –3.61 to –0.05, *p* = 0.044; 95% CI: –0.20 to –0.004, *p* = 0.039; 95% CI: –0.29 to –0.07, *p* = 0.001). The NT-proBNP, LVEDD, mPAP, PVR, and BDS values decreased in both cohorts (all *p* < 0.05), with more pronounced reductions in the experimental group (95% CI: 0.02–3.5, *p* = 0.048; 95% CI: 0.03–0.21, *p* = 0.012; 95% CI: 0.02–2.03, *p* = 0.046; 95% CI: 0.65–4.30, *p* = 0.008; 95% CI: 0.06–0.78, *p* = 0.022). The proportion of individuals in the WHO-FC classes III–IV was lower in the experimental group (95% CI: 1.05–4.56, *p* = 0.035). No statistically significant difference in adverse-event incidence was observed between groups (95% CI: 0.73–5.03, *p* = 0.184).

**Conclusion:**

Combination therapy with ambrisentan and riociguat effectively improved cardiopulmonary function and clinical outcomes in patients with high-risk coronary syndrome and PH, offering a promising therapeutic strategy for this population. This study is a single-center retrospective study, which inherently limits the credibility of causal inference; therefore, the results need to be further verified by multi-center, large-sample prospective studies.

## 1. Introduction

The primary physiological functions of the cardiovascular and respiratory systems are to maintain cellular respiration, and they are closely interconnected in both normal physiology and disease states. Cardiopulmonary structures are anatomically linked and functionally interdependent. Pulmonary disorders can influence cardiac function, and cardiovascular events may worsen pulmonary disease, thereby severely affecting clinical outcomes and prognosis. Coronary syndrome is among the most prevalent cardiovascular diseases and is categorized into chronic coronary syndrome (CCS) and acute coronary syndrome (ACS). It remains one of the leading causes of mortality in both men and women, contributing to approximately one-third of all global deaths [1, 2]. Coronary artery disease is characterized by lipid deposition within coronary artery walls, leading to progressive arterial stenosis and potentially complete vascular occlusion. Mortality increases with age and is higher in men, particularly between 35 and 55 years. After 55 years, male mortality tends to decline, whereas female mortality continues to rise, eventually reaching comparable levels beyond 70–75 years of age [3, 4]. Among these conditions, ACS warrants particular emphasis due to its severe threat to life and its position as a leading global cause of death. Pulmonary hypertension (PH) is a clinical and pathological syndrome defined by a mean pulmonary artery pressure (mPAP) exceeding 25 mmHg at rest or 30 mmHg during exercise, confirmed via right-heart catheterization. Clinically, PH may exist as an independent disease or develop secondary to diverse underlying conditions [5, 6]. Left-heart disease represents the predominant etiology of PH, accounting for approximately 65%–80% of cases. Once PH progresses to right-heart dysfunction, patients experience marked deterioration in symptoms and exercise tolerance, indicating a poor prognosis. Common left-heart conditions leading to PH include systolic heart failure (dilated cardiomyopathy, ischemic cardiomyopathy), diastolic dysfunction (hypertension, coronary atherosclerosis, hypertrophic cardiomyopathy), valvular heart disease, congenital or acquired left-heart inflow or outflow obstruction, hereditary cardiomyopathies, and selected congenital cardiac abnormalities (e.g., cor triatriatum) [7, 8, 9]. Early symptoms in patients with coronary artery disease complicated by PH are often subtle; however, disease progression results in severe manifestations such as dyspnea, angina, and syncope, accompanied by high disability and mortality. PH is frequently observed in older patients with coronary artery disease, and pathological analyses indicate a strong correlation between PH and left-heart dysfunction in this population [10].

Patients with coronary artery disease complicated by PH face greater therapeutic challenges compared with those without PH. Chronic pulmonary vascular congestion ultimately leads to sustained vasoconstriction, impaired nitric oxide (NO) synthesis, increased endothelin expression, and reduced sensitivity to B-type natriuretic peptide. These alterations result in progressive pulmonary vascular structural and functional remodeling, making targeted pharmacologic therapy a primary treatment strategy [9, 11]. Current targeted pharmacotherapies include endothelin receptor antagonists (ERAs), soluble guanylate cyclase agonists (sGCAs), phosphodiesterase-5 inhibitors (PDE5i), prostacyclin analogs, and prostacyclin receptor agonists. ERAs act by blocking endothelin receptors, mitigating vasoconstriction and cellular proliferation, reducing pulmonary arterial pressure, and inhibiting vascular remodeling. Common ERAs include bosentan, ambrisentan, and macitentan. Ambrisentan is a highly selective endothelin A receptor (ETA) antagonist that preserves vasodilatory NO and prostacyclin pathways mediated by the endothelin B receptor (ETB) [12]. A study by Qinhua Zhao *et al*. [13], incorporating five clinical trials, demonstrated similar efficacy between ambrisentan and bosentan, but highlighted better hepatic tolerance with ambrisentan in PH patients (hazard ratio (HR): 23.18; 95% confidence interval (CI): 2.24–377.20; surface under the cumulative ranking curve (SUCRA): ambrisentan 0.99, bosentan 0.02). sGCAs activate guanylate cyclase, increasing intracellular cyclic guanosine monophosphate (cGMP) and producing vasodilatory and anti-fibrotic effects. Key agents include riociguat and vericiguat. Riociguat is a novel sGCA with NO-independent and NO-enhanced dual activation mechanisms, thereby raising plasma cGMP, inducing vasodilation, reducing pulmonary vascular remodeling, and attenuating right-ventricular hypertrophy and fibrosis. A multi-center randomized open-label trial by Marius M. Hoeper *et al*. [14] demonstrated that riociguat may safely replace PDE5 inhibitors in PH patients with inadequate response and provide meaningful clinical benefit. Growing clinical evidence supports multi-drug regimens for PH. For instance, Ekkehard Grünig *et al*. [15] reported significant improvements in cardiac function and exercise capacity after 16 weeks of macitentan-tadalafil combination therapy in intermediate-to-high-risk PH patients. However, in high-risk coronary syndrome with PH, monotherapy often fails to achieve optimal outcomes, and improvements in survival and quality of life remain limited. Thus, evidence supporting multi-pathway pharmacologic synergy is essential.

This study evaluates the therapeutic effects of combined ERA and sGCA therapy (ambrisentan plus riociguat) in patients with high-risk coronary syndrome and PH, assessing improvements in exercise capacity, cardiopulmonary function, hemodynamics, and safety. It is anticipated that such combination therapy may provide multi-target benefits and more precise therapeutic effects for this complex patient population.

## 2. Materials and Methods

### 2.1 General Information

**Fig. 1.**
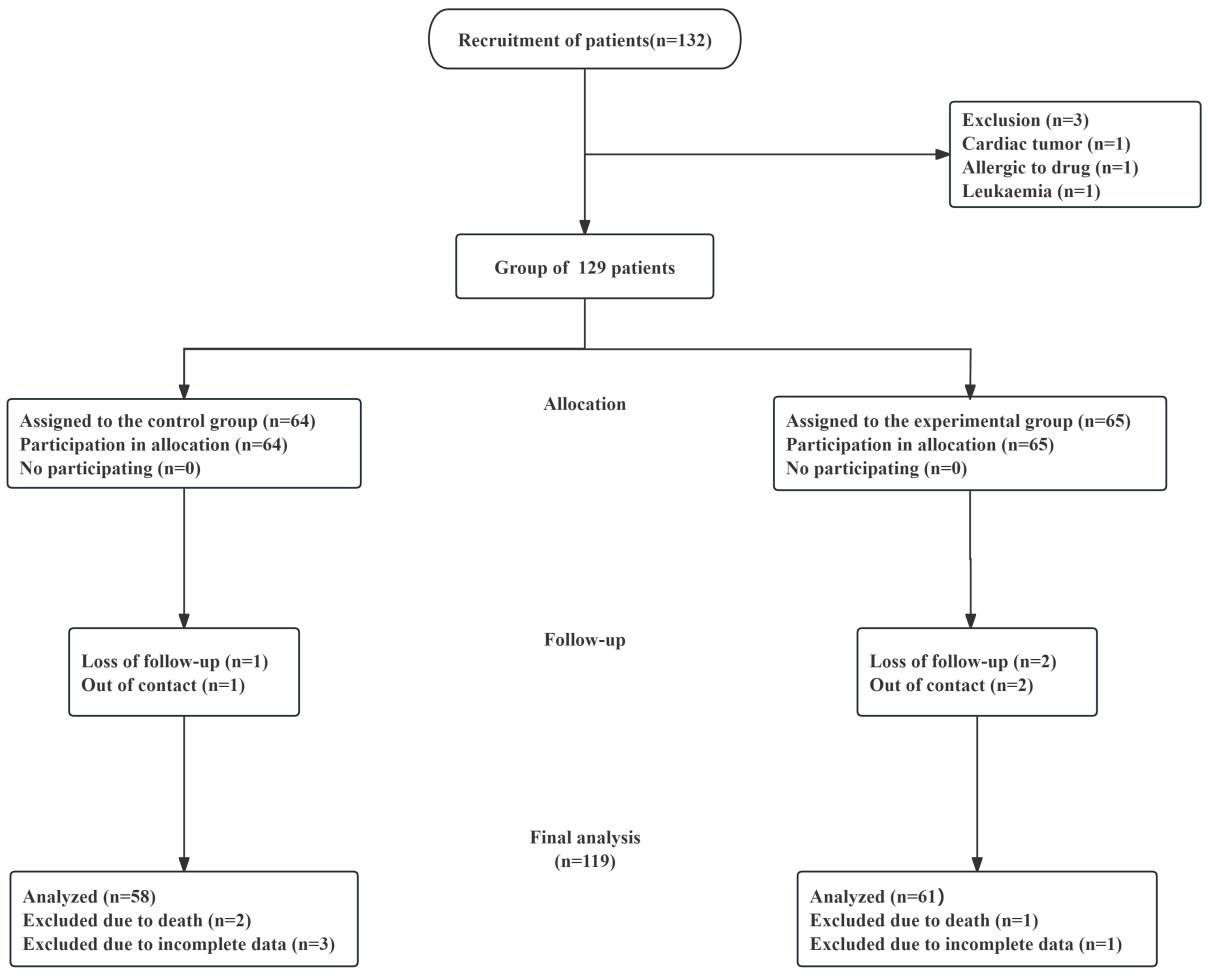
**Experimental process design diagram**. The flowchart depicts patient recruitment, inclusion, exclusion, and allocation procedures. Ultimately, 58 patients were allocated to the control group and 61 to the experimental group for comparative analysis.

This retrospective study included 132 patients with high-risk coronary syndrome complicated by PH who were admitted to the hospital between January 2019 and December 2023 to evaluate the efficacy of ambrisentan combined with riociguat. As illustrated in the study flowchart (Fig. 1), 129 patients remained after initial screening; among them, 3 patients were lost to follow-up, 3 died, and 4 were excluded due to incomplete data. Ultimately, a total of 119 patients were included in the final analysis and were categorized into the control group (ambrisentan monotherapy, n = 58) and the experimental group (ambrisentan plus riociguat, n = 61) according to their treatment strategy.

### 2.2 Inclusion Criteria

Patients were enrolled based on the following criteria:

(1) Meeting the diagnostic criteria for ACS according to the 2025 ACC/AHA/ACEP/NAEMSP/SCAI Guidelines for ACS evaluation and management;

(2) Cardiac troponin (cTn) levels exceeding the 99th percentile upper reference limit (URL) with >20% dynamic change;

(3) Presence of clinical manifestations of myocardial ischemia, including angina pectoris;

(4) Coronary angiography showing severe stenosis in major coronary supply territories (including proximal triple-vessel disease, particularly proximal left anterior descending artery (LAD) stenosis, or left main coronary artery disease);

(5) Coronary artery involvement ≥2 vessels;

(6) History of myocardial infarction and diabetes;

(7) New York Heart Association (NYHA) functional class ≥III;

(8) Positive Allen test results;

(9) PH confirmed by right-heart catheterization;

(10) Age between 35 and 75 years;

(11) Able to perform the 6-minute walk test with preserved basic motor function;

(12) Availability of complete clinical and examination data [16, 17].

### 2.3 Exclusion Criteria

Exclusion criteria were as follows:

(1) Presence of severe systemic diseases, such as malignancies or hematologic disorders;

(2) Severe hepatic or renal dysfunction;

(3) Other major cardiac conditions, including congenital heart disease, cardiomyopathy, or severe valvular disease;

(4) Ruptured sinus of Valsalva aneurysm or aortic dissection;

(5) Known hypersensitivity to study medications;

(6) Inability to tolerate long-term anticoagulation or antiplatelet therapy;

(7) Recent major surgery or significant trauma;

(8) History of psychiatric disorders [18].

### 2.4 Treatment Regimen

This investigation was a single-center, retrospective, observational, and controlled study. Treatment regimens were prescribed by attending physicians based on each patient’s clinical condition, comorbidities, medication tolerance, and current clinical guidelines. All patients underwent right-heart catheterization to confirm the diagnosis of PH following admission and prior to receiving percutaneous coronary intervention (PCI). High-risk coronary syndrome patients with PH were divided into two groups according to their pharmacological management plans. All patients received both interventional and pharmacotherapeutic treatment. PCI was performed using a digital subtraction angiography system (Innova 3100, GE Healthcare, Waukesha, WI, USA), with radial artery access for coronary angiography and intervention.

For medication therapy, all patients received routine antiplatelet therapy, including oral aspirin 100 mg (State Food and Drug Administration (SFDA) approval No. J20130078, Bayer Healthcare Co., Ltd., Leverkusen, Nordrhein-Westfalen, Germany) and a loading dose of 300 mg clopidogrel (SFDA approval No. J20180029, Sanofi S.A., Carbon Blanc, Nouvelle-Aquitaine, France) prior to PCI, followed by daily maintenance therapy with aspirin 100 mg and clopidogrel 75 mg after PCI [19].

Patients in the control group received conventional treatment along with ambrisentan (SFDA approval No. H20110023, Jiangsu Hansoh Pharmaceutical Co., Ltd., Lianyungang, Jiangsu, China), initiated at 5 mg once daily and increased to 10 mg once daily after 8 weeks based on tolerance and clinical response [20].

Patients in the experimental group received combination therapy consisting of ambrisentan (same regimen as the control group) plus riociguat (SFDA approval No. J20190001, Bayer Healthcare Co., Ltd., Leverkusen, North Rhine-Westphalia, Germany). Riociguat was initiated at 1 mg three times daily, with titration every 2 weeks according to tolerance, not exceeding a maximum of 2.5 mg three times daily [14].

Both groups received treatment for a total of 4 months. During therapy, clinical symptoms and laboratory findings were closely monitored, and drug dosages and therapeutic strategies were adjusted according to individual patient status.

### 2.5 Observation Indicators

#### 2.5.1 Primary Observation Indicators

2.5.1.1 Six-Minute Walk Distance (6MWD)

The 6MWD test required patients to walk as far as possible for 6 minutes along a flat 20-meter hospital corridor, and the total distance walked was recorded [21].

2.5.1.2 N-terminal pro-B-type Natriuretic Peptide (NT-proBNP Level)

Approximately 5 mL of fasting venous blood was collected into pro-coagulation tubes. Samples were centrifuged at 3000 r/min for 10 minutes using a centrifuge (Beckman Microfuge® 20R, Beckman Coulter, Brea, CA, USA) to separate serum, and NT-proBNP was measured using a human NT-proBNP ELISA kit (sensitivity: 0.216 ng/mL, CSB-E05152h, Huamei Biological, Wuhan, Hubei, China).

2.5.1.3 World Health Organization Functional Classification (WHO-FC)

The WHO-FC classification evaluates PH severity across four grades (I–IV).

• Class I: No limitation of ordinary physical activity; no symptoms with normal activity.

• Class II: Mild limitation of physical activity; no symptoms at rest but ordinary activity causes dyspnea, fatigue, chest pain, or near-syncope.

• Class III: Marked limitation of physical activity; no symptoms at rest but less-than-ordinary activity triggers symptoms.

• Class IV: Unable to carry out any physical activity without symptoms; symptoms present at rest, worsening with minimal exertion.

Cardiac function improvement was assessed by comparing proportions of patients in WHO-FC classes III and IV [22].

#### 2.5.2 Secondary Observation Indicators

2.5.2.1 Hemodynamic Parameters

Left ventricular end-diastolic diameter (LVEDD) and tricuspid annular plane systolic excursion (TAPSE) were evaluated using a cardiovascular ultrasound system (Recho R9, Mindray, Shenzhen, Guangdong, China). Cardiac index (CI), mPAP, and pulmonary vascular resistance (PVR) were assessed via right-heart catheterization.

2.5.2.2 Borg Dyspnea Score (BDS)

The BDS ranges from 0–10 to quantify exertional dyspnea, where 10 reflects intolerable breathlessness and 0 represents no dyspnea. Higher scores indicate worse exercise tolerance.

2.5.2.3 Incidence of Adverse Events

Adverse events during ambrisentan and riociguat therapy included fluid retention/edema, nasal congestion, worsening hypoxemia, and acute kidney injury. The incidence of adverse reactions was recorded [23].

### 2.6 Sample Size Calculation

Sample-size estimation was conducted using G*Power (3.1.9.7, Heinrich-Heine-Universität Düsseldorf, Düsseldorf, North Rhine-Westfalen, Germany). Based on the study by Theresa Marie Dachs *et al*. [24] in PH patients with heart failure, an effect size of 0.31 was employed, demonstrating clinical significance. With α = 0.05 (two-sided) and power (1-β) = 0.80, the estimated required sample was 44 participants per group (88 total). Accounting for a 20% dropout rate, a minimum of 114 participants was required. Ultimately, 58 control-group patients and 61 experimental-group patients (119 total) were included, providing adequate statistical power for reliable inference.

### 2.7 Statistical Methods

Statistical analyses were performed using SPSS 26 (IBM Corporation, Armonk, NY, USA). Normally distributed continuous variables (e.g., 6MWD, NT-proBNP, hemodynamic parameters, BDS) were reported as mean ± standard deviation (±s). Between-group comparisons used independent-samples *t*-tests, and paired-samples *t*-tests assessed within-group pre- vs post-treatment changes. Non-normally distributed data were presented as median (interquartile range) [M (IQR)], with Mann-Whitney U tests for between-group comparisons. Categorical variables (e.g., WHO-FC, adverse events, baseline characteristics) were reported as n (%) and compared using chi-square tests. Statistical significance was set at *p* < 0.05.

## 3. Results

### 3.1 Comparison of Baseline Data Between the Two Groups

As presented in Table 1, baseline characteristics of the two patient groups were compared, including age, body mass index (BMI), gender, heart rate (HR), smoking status, alcohol consumption history, NYHA functional classification, presence of left main coronary artery lesions, proportion of three-vessel disease, ACS subtypes, Killip class, and left ventricular ejection fraction (LVEF). There were no statistically significant differences between the control and experimental groups across these variables (all *p* > 0.05), indicating that the two groups were well-matched and comparable prior to treatment.

**Table 1.** **Baseline characteristics [±*s*, *n* (%)]**.

Variables		Control group (n = 58)	Experimental group (n = 61)	95% CI of the difference		*p*	Effect size
				Lower	Upper		
Age (years)		57.38 ± 10.77	58.21 ± 9.12	–4.45	2.78	0.649	Cohen’ D = –0.080
BMI (kg/m^2^)		23.98 ± 1.60	23.47 ± 1.72	–0.09	1.12	0.093	Cohen’ D = 0.310
Gender							
	Male	32 (55.17)	33 (54.10)			0.906	Phi = 0.011
	Female	26 (44.83)	28 (45.90)				
HR (bpm)		70.00 ± 5.54	70.02 ± 5.41	–2.00	1.97	0.987	
Smoking		31 (53.45)	29 (47.54)			0.519	Phi = 0.059
Drinking		35 (60.34)	37 (60.66)			0.972	Phi = –0.003
NYHA-FC							
	III	33 (56.90)	35 (57.38)			0.958	Phi = –0.005
	IV	25 (43.10)	26 (42.62)				
Left main coronary artery		36 (62.07)	38 (62.30)			0.980	Phi = –0.002
Coronary artery three-ressel disease		32 (55.17)	36 (59.02)			0.672	Phi = –0.039
ACS subtypes							
	STEMI	21 (36.21)	23 (37.70)			0.979	Cramer’s V = 0.019
	NSTEMI	18 (31.03)	19 (31.15)				
	UA	19 (32.76)	19 (31.15)				
Killip class							
	II	24 (41.38)	26 (42.62)			0.989	Cramer’s V = 0.014
	III	28 (48.28)	29 (47.54)				
	IV	6 (10.34)	6 (9.84)				
EF (%)		30.98 ± 2.19	31.22 ± 2.08	–1.02	0.53	0.538	Cohen’ D = –0.113

Note: BMI, Body mass index; HR, Heart rate; NYHA-FC, New York Heart Association functional classification; ACS, Acute Coronary Syndrome; STEMI, ST-segment elevation myocardial infarction; NSTEMI, Non-ST-segment elevation myocardial infarction; UA, Unstable angina; EF, Ejection fraction; CI, Confidence Interval.

### 3.2 Comparison of 6MWD Between the Two Groups

The 6MWD was compared between the control and experimental groups, and the results are presented in Table 2. Prior to treatment, no statistically significant difference in 6MWD was observed between the two groups (95% CI: –1.64–2.00; *p* = 0.844). Following treatment, 6MWD increased significantly in both groups (all *p* < 0.05), indicating substantial improvement in exercise tolerance in all patients. However, the improvement was significantly greater in the experimental group compared with the control group (524.62 ± 4.90 m vs. 522.79 ± 4.88 m; 95% CI: –3.61 to –0.05; *p* = 0.044). These results suggest that combination therapy with ambrisentan and riociguat is more effective in enhancing exercise tolerance than ambrisentan monotherapy.

**Table 2.** **Comparison of 6MWD of patients (±*s*, m)**.

Variables		6MWD	
Time		Before treatment	After treatment
Control group (n = 58)		500.67 ± 5.03	522.79 ± 4.88*
Experimental group (n = 61)		500.49 ± 4.97	524.62 ± 4.90*
95% CI of the difference	Lower	–1.64	–3.61
	Upper	2.00	–0.05
*p*		0.844	0.044
Effect size (Cohen’ D)		0.04	–0.37

Note: *p < 0.05 vs. Before treatment; 6MWD, 6-minute walk distance.

### 3.3 Comparison of NT-proBNP Levels Between the Two Groups

As shown in Table 3, there was no statistically significant difference in NT-proBNP levels between the two groups prior to treatment (95% CI: –3.29–0.18; *p* = 0.079). Following treatment, NT-proBNP levels decreased significantly in both groups (all *p* < 0.05), indicating improvement in cardiac function. Furthermore, post-treatment NT-proBNP levels in the experimental group (724.62 ± 4.90 pg/mL) were significantly lower than those in the control group (726.38 ± 4.67 pg/mL) (95% CI: 0.02–3.5; *p* = 0.048). These findings suggest that while both treatment regimens alleviated heart failure severity, the combination of ambrisentan and riociguat resulted in a more pronounced reduction in NT-proBNP levels compared with ambrisentan monotherapy.

**Table 3.** **Comparison of NT-proBNP of patients (±*s*, pg/mL)**.

Variables		NT-proBNP	
Time		Before treatment	After treatment
Control group (n = 58)		1523.07 ± 4.64	726.38 ± 4.67*
Experimental group (n = 61)		1524.62 ± 4.90	724.62 ± 4.90*
95% CI of the difference	Lower	–3.29	0.02
	Upper	0.18	3.50
*p*		0.079	0.048
Effect size (Cohen’ D)		–0.32	0.37

Note: *p < 0.05 vs. Before treatment. NT-proBNP, N-terminal pro-B-type natriuretic peptide.

### 3.4 Comparison of WHO-FC Between the Two Groups

As shown in Table 4, prior to treatment, the control group included 33 patients in WHO-FC class III and 25 in class IV (total n = 58), while the experimental group consisted of 35 patients in class III and 26 in class IV (total n = 61). Following treatment, the control group included 25 patients in WHO-FC class III and 10 in class IV (total n = 35), representing 60.3% of the cohort. In the experimental group, 14 patients were in WHO-FC class III and 11 in class IV (total n = 25), accounting for 41% of the cohort. These results indicate that the proportion of patients in WHO-FC classes III and IV decreased in both groups after treatment, with a more pronounced reduction observed in the experimental group (95% CI: 1.05–4.56; *p* = 0.035). Overall, these findings suggest improvement in cardiac functional status in both groups, with combination therapy using ambrisentan and riociguat resulting in greater improvement compared with ambrisentan alone.

**Table 4.** **Comparison of WHO-FC of patients [*n (%)*]**.

Indicator		WHO-FC			
Time		Before treatment		After treatment	
Group		Control group (n = 58)	Experimental group (n = 61)	Control group (n = 58)	Experimental group (n = 61)
	I	-	-	2 (3.5)	6 (9.8)
	II	-	-	21 (36.2)	30 (49.2)
	III	33 (56.9)	35 (57.4)	25 (43.1)	14 (23.0)
	IV	25 (43.1)	26 (42.6)	10 (17.2)	11 (18.0)
	Ⅲ + IV	58 (100)	61 (100)	35 (60.3)*	25 (41.0)*
*p*		-	-	0.035	-
Effect size (Cramer’s V)		-	-	0.194	-

Note: *p < 0.05 vs. Before treatment; WHO-FC, World Health Organization functional classification.

### 3.5 Comparison of TAPSE and LVEDD Between the Two Groups

As shown in Table 5, no significant differences in TAPSE and LVEDD were observed between the two groups before treatment (95% CI: –0.09–0.08, *p* = 0.929; 95% CI: –0.06–0.12, *p* = 0.467). Following treatment, TAPSE increased and LVEDD decreased in both groups (all *p* < 0.05), indicating improvement in right and left ventricular function. Moreover, compared with the control group (TAPSE: 17.71 ± 0.26 mm; LVEDD: 51.49 ± 0.25 mm), the experimental group demonstrated a significantly greater increase in TAPSE (17.90 ± 0.34 mm; 95% CI: –0.29 to –0.07, *p* = 0.001) and a more significant reduction in LVEDD (51.37 ± 0.24 mm; 95% CI: 0.03–0.21, *p* = 0.012). These findings indicate that combination therapy with ambrisentan and riociguat provides superior improvement in both left and right ventricular function compared with ambrisentan monotherapy.

**Table 5.** **Comparison of TAPSE/LVEDD of patients (±*s*, mm)**.

Variables		TAPSE		LVEDD	
Time		Before treatment	After treatment	Before treatment	After treatment
Control group (n = 58)		16.52 ± 0.24	17.71 ± 0.26*	54.56 ± 0.25	51.49 ± 0.25*
Experimental group (n = 61)		16.52 ± 0.24	17.90 ± 0.34*	54.52 ± 0.24	51.37 ± 0.24*
95% CI of the difference	Lower	–0.09	–0.29	–0.06	0.03
	Upper	0.08	–0.07	0.12	0.21
*p*		0.929	0.001	0.467	0.012
Effect size (Cohen’ D)		0	–0.63	0.16	0.49

Note: *p < 0.05 vs. Before treatment; LVEDD, Left ventricular end-diastolic diameter; TAPSE, Tricuspid annular plane systolic excursion.

### 3.6 Comparison of mPAP and PVR Between the Two Groups

According to the data in Table 6, no significant differences were observed between the two groups in mPAP and PVR before treatment (95% CI: –0.96–1.02, *p* = 0.948; 95% CI: –1.64–2.00, *p* = 0.844). After treatment, both mPAP and PVR were significantly reduced in both groups (all *p* < 0.05), suggesting a mitigation of pulmonary hypertension. Furthermore, post-treatment values in the experimental group (mPAP: 19.15 ± 2.85 mmHg; PVR: 255.89 ± 4.90 dyn⋅s⋅cm^-5^) were significantly lower than those in the control group (mPAP: 20.17 ± 2.70 mmHg; PVR: 258.36 ± 5.17 dyn⋅s⋅cm^-5^) (95% CI: 0.02–2.03, *p* = 0.046; 95% CI: 0.65–4.30, *p* = 0.008). These results indicate that pulmonary hypertension was alleviated in both groups, and the combined use of ambrisentan and riociguat resulted in a more pronounced improvement than ambrisentan monotherapy.

**Table 6.** **Comparison of mPAP/PVR of patients (±*s*)**.

Variables		mPAP (mmHg)		PVR (dyn·s·cm^–⁢5^)	
Time		Before treatment	After treatment	Before treatment	After treatment
Control group (n = 58)		25.00 ± 2.75	20.17 ± 2.70*	500.67 ± 5.03	258.36 ± 5.17*
Experimental group (n = 61)		24.97 ± 2.70	19.15 ± 2.85*	500.49 ± 4.97	255.89 ± 4.90*
95% CI of the difference	Lower	–0.96	0.02	–1.64	0.65
	Upper	1.02	2.03	2.00	4.30
*p*		0.948	0.046	0.844	0.008
Effect size (Cohen’ D)		0.01	0.37	0.04	0.49

Note: *p < 0.05 vs. Before treatment; mPAP, Mean pulmonary artery pressure; PVR, Pulmonary vascular resistance.

### 3.7 Comparison of CI Between the Two Groups

As presented in Table 7, no statistically significant difference in CI was observed between the control and experimental groups prior to treatment (95% CI: –0.10–0.10, *p* = 0.969). Following treatment, CI increased significantly in both groups (all *p* < 0.05), with a more marked improvement observed in the experimental group compared with the control group (3.09 ± 0.26 L/min/m^2^ vs. 3.20 ± 0.27 L/min/m^2^; 95% CI: –0.20 to –0.004, *p* = 0.039). These results indicate that combination therapy with ambrisentan and riociguat more effectively enhances cardiac pumping efficiency, thereby improving perfusion and alleviating ischemia and hypoxia in critical peripheral organs.

**Table 7.** **Comparison of CI of patients (±*s*, L/min/m^2^)**.

Variables		CI	
Time		Before treatment	After treatment
Control group (n = 58)		2.49 ± 0.26	3.09 ± 0.26*
Experimental group (n = 61)		2.50 ± 0.27	3.20 ± 0.27*
95% CI of the difference	Lower	–0.10	–0.20
	Upper	0.10	–0.004
*p*		0.969	0.039
Effect size (Cohen’ D)		–0.04	–0.42

Note: *p < 0.05 vs. Before treatment; CI, cardiac index.

### 3.8 Comparison of BDS Between the Two Groups

As presented in Table 8, there was no significant difference in BDS between the two groups prior to treatment (95% CI: –0.51–0.46, *p* = 0.929). Following treatment, BDS decreased significantly in both groups (all *p* < 0.05). Moreover, the experimental group demonstrated a significantly lower post-treatment BDS (1.75 ± 0.98) compared with the control group (2.17 ± 0.99) (95% CI: 0.06–0.78, *p* = 0.022). These findings suggest that dyspnea symptoms improved in both patient groups after treatment, with the combination of ambrisentan and riociguat resulting in a more substantial improvement than ambrisentan monotherapy.

**Table 8.** **Comparison of BDS of patients (±*s*, score)**.

Variables		BDS	
Time		Before treatment	After treatment
Control group (n = 58)		2.55 ± 1.24	2.17 ± 0.99*
Experimental group (n = 61)		2.57 ± 1.42	1.75 ± 0.98*
95% CI of the difference	Lower	–0.51	0.06
	Upper	0.46	0.78
*p*		0.929	0.022
Effect size (Cohen’ D)		–0.02	0.43

Note: *p < 0.05 vs. Before treatment; BDS, Borg dyspnea score.

### 3.9 Comparison of Adverse Event Incidence Between the Two Groups

Table 9 summarizes the incidence of adverse events observed during treatment. In the control group, there were 6 cases of fluid retention/edema, 3 cases of sinus congestion, 2 cases of worsening hypoxemia, and 2 cases of acute kidney injury, resulting in an overall adverse event rate of 22.4%. In the experimental group, there were 4 cases of fluid retention/edema, 2 cases of sinus congestion, and 2 cases of worsening hypoxemia, with a total adverse event rate of 13.1%. These findings indicated that there was no statistically significant difference in the incidence of adverse events between the ambrisentan monotherapy group and the combination therapy group (95% CI: 0.73–5.03, *p* = 0.184).

**Table 9.** **Comparison of adverse events of patients [*n* (%)]**.

Variables	Adverse events				
	fluid retention/edema	sinus congestion	worsening hypoxemia	acute kidney injury	total
Control group (n = 58)	6 (10.3)	3 (5.2)	2 (3.4)	2 (3.4)	13 (22.4)
Experimental group (n = 61)	4 (6.6)	2 (3.3)	2 (3.3)	0 (0.0)	8 (13.1)
*p*	0.184				
Effect size (Cramer’s V)	0.122				

## 4. Discussion

Coronary syndrome results from stenosis or occlusion of the coronary vascular lumen secondary to atherosclerosis, leading to myocardial ischemia and hypoxia and ultimately impairing cardiac pump function, which poses a serious threat to patient survival. Patients with coronary syndrome complicated by PH generally exhibit higher mortality rates. Previous studies have reported that the morbidity of chronic heart failure complicated by PH is approximately 2%, with incidence increasing with age, thereby elevating mortality risk in middle-aged and elderly individuals and imposing a considerable socioeconomic burden on families and healthcare systems [25]. Coronary syndrome and PH share intertwined pathophysiological mechanisms, making management challenging. With advances in understanding the interaction between these two conditions, combination pharmacotherapy has emerged as a promising therapeutic concept, aiming to optimize coronary blood flow, reduce right ventricular (RV) load, improve myocardial metabolism, and minimize adverse drug interactions [26].

The present study focused on high-risk coronary syndrome patients with PH. Coronary artery stenosis in these patients leads to myocardial ischemia. During exertion, myocardial oxygen demand increases, yet coronary blood flow fails to adequately rise, resulting in impaired left ventricle (LV) systolic and diastolic function and limited cardiac output (CO). Concurrently, PH causes sustained elevation in RV afterload; PVR further increases during exertion, impairing RV ejection and reducing RV output. RV dilation may also compress the LV, limiting LV filling and subsequently diminishing CO, contributing to reduced exercise tolerance [27, 28]. Compared with coronary artery disease patients without PH, those with concomitant PH present with worse cardiac function, higher NYHA class III/IV proportion, greater LVEDD and left atrial (LA) enlargement, reduced LVEF, and elevated natriuretic peptides.

Our findings are consistent with previous studies. Hossein-Ardeschir Ghofrani *et al*. [29], in the PATENT study, demonstrated good tolerability and clinical benefit with long-term ERA plus riociguat therapy, including a 24-meter increase in 6MWD and WHO-FC improvement in 57% of patients. In our study, the combination group similarly showed a significant increase in 6MWD and a decrease in WHO-FC class III/IV patients. A meta-analysis by Mustafa Erdogan *et al*. [30], including pharmacologic agents such as sildenafil, bosentan, ambrisentan, and riociguat, demonstrated reductions in NT-proBNP and improvements in CI, aligning with our results, where NT-proBNP decreased significantly and CI increased after therapy.

Previous literature investigating ERA-based combination strategies reported similar trends. Qinhua Zhao *et al*. [31] demonstrated significant TAPSE improvement with ERA and PDE5i combination (*p* = 0.006). Likewise, in a prospective single-arm open-label study by Jason Weatherald *et al*. [32], PVR decreased by 54% at 4 months with ambrisentan-riociguat therapy. Furthermore, Panagiota Xanthouli *et al*. [33] in the EDITA study observed significant mPAP reductions in ambrisentan-treated patients. In our cohort, LVEDD, PVR, and mPAP decreased, whereas TAPSE increased, consistent with these findings.

These physiological improvements likely stem from the pathophysiology of PH in coronary artery disease: PH enlarges LV dimensions, reduces LV systolic function, and impairs oxygen delivery, exacerbating pulmonary congestion and ventilation–perfusion imbalance. Elevated RV pressure and morphological RV changes further compromise LV filling and perfusion. Mechanistically, riociguat activates soluble guanylate cyclase (sGC), increases cGMP production independent of NO, dilates pulmonary vasculature, reduces PVR, and suppresses smooth muscle proliferation and fibrosis [34]. Ambrisentan selectively antagonizes endothelin-1 (ET-1) ETA receptors, attenuating vasoconstriction, reducing vascular remodeling, and lowering mPAP. Acting on complementary pathways, their combination yields synergistic vasodilatory and anti-remodeling effects, markedly reducing PVR and mPAP and enhancing CO more effectively than monotherapy.

Ambrisentan blocks the vasoconstrictive pathway mediated by ET-1, while riociguat enhances vasodilatory effects via the cGMP pathway. Together, they regulate vascular tone in both the pulmonary and coronary circulations, significantly reducing PVR and mPAP. Meanwhile, ambrisentan inhibits the proliferation of cardiomyocytes and fibroblasts, and riociguat reduces collagen deposition through the cGMP pathway. These two drugs synergistically alleviate myocardial fibrosis and pulmonary vascular remodeling, thereby improving LVEDD and CI. Excessive ET-1 leads to oxidative stress and apoptosis of cardiomyocytes, and ambrisentan can reduce the production of oxidative stress products by blocking receptors. The cGMP pathway, on the other hand, can activate anti-apoptotic signals; riociguat increases cGMP levels to further inhibit cardiomyocyte apoptosis. Together, they protect myocardial function, improve TAPSE, and enhance WHO-FC. Pulmonary vascular remodeling caused by PH impairs gas exchange. The synergistic reduction of PVR by the two drugs improves pulmonary perfusion. Additionally, the cGMP pathway inhibits airway smooth muscle contraction, and ambrisentan reduces ET-1-mediated airway inflammatory responses. These combined effects decrease the BDS score and increase the 6MWD.

Additionally, no statistically significant difference was observed in adverse-event incidence between groups in our study. Consistent with Raziye Ceylan *et al*. [35], who reported an association between PH and dyspnea (r = –0.468, *p* < 0.05), our findings demonstrated significant reductions in BDS and dyspnea severity in the combination group. Through multi-target modulation, riociguat and ambrisentan decrease PVR, improve RV function, optimize ventilation–perfusion balance, and reduce respiratory muscle load, thereby interrupting the vicious pathophysiologic cycle of PH-related dyspnea. This translates not only to symptom relief at rest but also enhanced exercise endurance and improved quality of life, supporting the clinical value of combination therapy in high-risk coronary syndrome patients with PH.

## 5. Limitations

This study adopted a retrospective design. Although data collection and analysis were conducted by investigators not involved in patient management, thereby ensuring a certain degree of objectivity, retrospective research inherently carries methodological limitations and limits the credibility of causal inference. The 119 enrolled patients were all from a single center. Despite meeting the basic statistical requirements, the sample size remained relatively small and did not adequately represent populations of diverse socioeconomic and cultural backgrounds, potentially introducing selection bias and limiting the generalizability of the findings. Furthermore, coronary syndrome encompasses both acute and chronic presentations, yet patients in this study were not stratified according to these subtypes, which may have influenced subgroup interpretation.

Another limitation lies in the primary focus on short-term therapeutic outcomes, without in-depth evaluation of long-term prognosis or quality-of-life improvements. Given the complexity of high-risk coronary syndrome combined with PH, long-term cardiopulmonary functional changes, disease progression, and patient-reported outcomes are essential in determining the full clinical value of combination therapy. Additionally, documentation of adverse drug reactions was not sufficiently comprehensive, and rare but clinically significant adverse events may have gone undetected due to the limited sample size and short follow-up duration.

Future studies should employ multicenter, large-scale, prospective randomized controlled trial designs to expand the sample size and include populations from different regions and backgrounds, thereby enhancing the universality and reliability of results. Furthermore, long-term follow-up and stratified randomization (including refined coronary syndrome classification) are recommended to provide more rigorous evidence and stronger theoretical support for clinical practice.

## 6. Conclusion

In summary, dual-drug therapy for high-risk coronary syndrome complicated by PH demonstrates clear therapeutic advantages by concurrently improving coronary perfusion, reducing pulmonary circulation pressure, and protecting cardiac function through multi-target synergistic mechanisms. This approach addresses limitations associated with monotherapy and contributes to improved patient quality of life. With continued advancement in the understanding of comorbidity mechanisms, combination therapy strategies are expected to become increasingly precise and individualized, offering more effective therapeutic options for this complex patient population.

## Availability of Data and Materials

The datasets generated during and analysed during the current study are available from the corresponding author on reasonable request.
